# Copper-Doped Bioactive Glass as Filler for PMMA-Based Bone Cements: Morphological, Mechanical, Reactivity, and Preliminary Antibacterial Characterization

**DOI:** 10.3390/ma11060961

**Published:** 2018-06-06

**Authors:** Marta Miola, Andrea Cochis, Ajay Kumar, Carla Renata Arciola, Lia Rimondini, Enrica Verné

**Affiliations:** 1 Department of Applied Science and Technology, Politecnico di Torino, 10129 Torino, Italy; marta.miola@polito.it; 2 Department of Health Sciences, Università del Piemonte Orientale UPO, 28100 Novara, Italy; andrea.cochis@med.uniupo.it (A.C.); ajaykumar2420@gmail.com (A.K.); lia.rimondini@med.uniupo.it (L.R.); 3 Interdisciplinary Research Center of Autoimmune Diseases (IRCAD), 28100 Novara, Italy; 4 Research Unit on Implant Infections, Rizzoli Orthopaedic Institute, 40136 Bologna, Italy; carlarenata.arciola@ior.it; 5 Department of Experimental, Diagnostic and Specialty Medicine, University of Bologna, 40126 Bologna, Italy

**Keywords:** copper, bioactive glass, antimicrobial, composite bone cement

## Abstract

To promote osteointegration and simultaneously limit bacterial contamination without using antibiotics, we designed innovative composite cements containing copper (Cu)-doped bioactive glass powders. Cu-doped glass powders were produced by a melt and quenching process, followed by an ion-exchange process in a Cu salt aqueous solution. Cu-doped glass was incorporated into commercial polymethyl methacrylate (PMMA)-based cements with different viscosities. The realized composites were characterized in terms of morphology, composition, leaching ability, bioactivity, mechanical, and antibacterial properties. Glass powders appeared well distributed and exposed on the PMMA surface. Composite cements showed good bioactivity, evidencing hydroxyapatite precipitation on the sample surfaces after seven days of immersion in simulated body fluid. The leaching test demonstrated that composite cements released a significant amount of copper, with a noticeable antibacterial effect toward *Staphylococcus epidermidis* strain. Thus, the proposed materials represent an innovative and multifunctional tool for orthopedic prostheses fixation, temporary prostheses, and spinal surgery.

## 1. Introduction

Hospital-acquired infections are the third-largest cause of public health problems. According to the most recent surveys, the mortality rate of patients experiencing infections after undergoing primary implant ranges from 10% to 18% [1]. Moreover, if a further infection occurs in the revised implants, these percentages can double or triple [2]. Most infections are due to a group of multi-drug resistant (MDR) pathogenic biofilm producer strains that are resistant to common antibiotic therapies. So, a huge need for new antibacterial active agents exists that are able to overcome bacteria resistance to antibiotics. Among the many alternative antibacterial compounds, copper is an interesting tool due to its ability to kill bacteria through the “contact killing” mechanism [3]. Through this mechanism, after release, copper is largely accumulated in the intracellular microenvironment. Here, exposure to copper leads to progressive membrane damage that causes bacterial death [3]. The involved pathways are not yet completely understood. An initial hypothesis is related to the huge oxidative stress induced by copper accumulation. The oxidative stress arises from the redox cycling between Cu, Cu (I), and Cu (II) [4]. Moreover, aerobic conditions enable copper to produce hydroxyl radicals according to the Haber-Weiss and Fenton reactions [5]. However, this hypothesis does not always hold true. Weaver et al. reported the fast-killing activity of copper toward a MDR *S. aureus* strain, but membrane damage was not evident [6]. So, the authors hypothesized an alternative mechanism of contact killing based on the triggering of reactive oxygen species (ROS) production and a subsequent arrest in cellular respiration responsible for a lethal DNA damage. Another interesting antibacterial copper activity was discovered by Macomber and Imlay [7]. They suggested that copper ions can determine the displacement of iron atoms from iron-sulphur clusters by linking to the same sulphur atoms. The rapid copper inactivation of iron-sulphur cluster enzymes results in damaging several central catabolic and biosynthetic pathways [7]. Copper is also used for drinking water treatment and in air conditioning systems; it was the first inorganic antimicrobial agent recognized by the American Environmental Protection Agency in 2008 [8,9].

Copper-containing biomaterials, both for hard and soft tissues, have drawn the attention of researchers [10,11,12,13,14,15] due to the angiogenetic and antimicrobial properties of copper [8,9,10,15,16,17]. The ability to modify the composition of bioactive glasses by introducing therapeutic ions has been exploited to create copper-containing bioactive devices [18]. Cu-containing bioactive glasses have mainly been prepared using the sol-gel process [10,12,13,19,20] and the traditional melt and quenching process [11,15,16,21], whereas only few studies used the ion-exchange technique to introduce Cu into the glass composition. For the first time, the process of introducing Cu ions into bioactive glasses with the ion-exchange technique in molten salts in aqueous solution was reported [22]. These studies demonstrated that Cu-loaded glasses are able to limit bacterial adhesion and proliferation, thus reducing the risk of infection development [10,11,12].

Polymethyl methacrylate (PMMA)-based bone cements are widely used as bone filler and for the fixation of prosthetic devices [23]. However, this material is not bioactive, creates a weak mechanical bond with host tissues, and is prone to bacterial contamination [24]. To overcome these drawbacks, several additives were incorporated into the polymeric matrix [25]. In particular, bioactive glasses and ceramics were used as fillers to promote material osteointegration [26,27], where antibiotics were incorporated into PMMA-based cement to reduce the development of infection [28]. However, the prolonged use of antibiotic-loaded bone cement resulted in the development of resistant bacteria [28,29]. So, the scientific community studied new approaches to develop antibacterial bone cements. PMMA-based bone cement containing silver micro- and nano-particles were investigated [30,31,32,33,34]. Authors demonstrated that silver-loaded bone cements possess in vitro antimicrobial properties and that the presence of silver does not affect bone cement properties, such as biocompatibility and mechanical properties. Nevertheless, Moojen et al. stated that bone cements containing silver (Ag) nanoparticles were not effective in preventing in vivo infections [35]. Authors proposed an innovative solution by incorporating bioactive and Ag-doped glass powders in PMMA bone cements, thus creating a composite bone cement that is also bioactive and antibacterial without the use of antibiotics [36,37,38,39]. Furthermore, the addition of a single phase, having both bioactive and antibacterial properties, allowed the maintenance of mechanical properties.

The aim of this study was to synthesize composite bone cements containing a bioactive glass doped with copper ions and to characterize the cements in terms of morphology, composition, leaching ability, bioactivity, and mechanical and antibacterial properties. To the best of our knowledge, this is the first time that a copper-containing bioactive glass was used as a filler in PMMA-based bone cements. Copper was selected for its lower toxicity and higher cytocompatibility with respect to silver and for its ability to be metabolized [40]. In a previous work [22], we developed the first bioactive and antibacterial Cu-doped glass via the ion-exchange process. In the present study, we investigated the possibility of using optimized glass as a filler in PMMA-based bone cements, balancing the amount of glass to maintain the mechanical properties of the cements and allowing glass particles to be exposed on the composite surface to impart bioactive and antibacterial properties.

## 2. Materials and Methods

### 2.1. Synthesis of Glass and Composite Cements

In this work, a bioactive glass with the following molar composition was synthesized by means of melt and quenching technique by melting the reactants in a Pt crucible at 1450 °C for 1 h: 48% SiO_2_, 26% Na_2_O, 22% CaO, 3% P_2_O_5_, 0.43% B_2_O_3_, and 0.57% Al_2_O_3_ (SBA3). Then, the melt was quenched in distilled water to obtain a frit, which was ball milled and sieved to obtain powders <20 µm [22]. Subsequently, SBA3 powders were ion-exchanged in a copper-containing aqueous solution (0.05 M) for 1 h at 37 °C to allow the introduction of copper in the glass network at the expense of modifier ions (Na^+^ and Ca^++^), as previously reported [22]. At the end of the process, the powders (Cu-SBA3) were gently washed twice with bi-distilled water, filtered using disk filter paper, and dried at 60 °C for 12 h. Cu-SBA3 powders, previously passed through a pestle to remove agglomerates due to the drying process, were then used as a filler (10 wt %) in commercial acrylic bone cements (CEMEX^®^, Tecres S.p.A., Sommacampagna (VR) Italy) with different viscosities, as reported in Table 1. Cements having different viscosities were analysed in terms of glass distribution, mechanical properties, bioactivity, and copper release. Commercial Cemex^®^ bone cements include a kit containing a sachet of solid phase (spherical pre-polymerized PMMA, BaSO_4_ as a radio-opaque agent, and benzoyl peroxide as an initiator) and an ampoule of liquid containing the monomer (methyl methacrylate; MMA), *N*,*N*-dimethyl-toluidine as the activator, and hydroquinone as the inhibitor. Composite bone cements were prepared by mechanically mixing Cu-SBA3 powders with the solid phase of commercial cement for 1 h to reach a good dispersion of glass in the pre-polymerized PMMA. Subsequently, the liquid phase was added to mixed powders, using the same ratio (solid/liquid phase) of commercial products as reported in Table 1. The mixture was stirred according to manufacturer instruction (about 1–1.5 min) and, as soon as the paste did not stick to the gloves, it was inserted into a polished aluminium mold with dimensions useful for further characterizations.

### 2.2. Composite Bone Cements Characterization

Copper doped glass was completely characterized in a previous work [24]. In this paper, composite bone cements were characterized in terms of morphology, composition, in vitro reactivity in simulated body fluid (SBF, Kokubo [41]), compressive strength, and copper release. All composite bone cements (CEMEX^®^ ISO-Cu, CEMEX^®^ RX-Cu, and CEMEX^®^ XL-Cu) were subjected to morphological-compositional analyses by means of field emission scanning electron microscopy (FESEM, SUPRATM 40, Zeiss, Oberkochen, Germany) equipped with energy dispersive X-ray spectroscopy (EDS) (Oxford Instruments, Abingdon, Oxfordshire, UK). To perform the measurement, cylindrical specimens (5 and 10 mm in diameter) were attached to aluminium (Al) stubs with a silver-based glue and metallized with chromium (Cr). The bioactivity of the composites and the ability to promote the precipitation of hydroxyapatite (HAp) on their surface were estimated by dipping samples (3 samples for each viscosity and incubation time) in 30 mL of SBF at 37 °C for up to one month. The solution was refreshed every 2–3 days to mimic the natural renewal of physiological fluids and the pH was measured at every refresh. After 7, 14, and 28 days of immersion, samples were removed from the solution, gently washed in bi-distilled water, and dried at room temperature overnight. Then, samples were subjected to FESEM-EDS analyses to estimate the precipitation of HAp or its precursors on their surface. Since the introduction of an additional phase in the polymeric matrix can reduce the mechanical properties, a preliminary mechanical characterization of composite cements was performed by evaluating their compressive strength compared to commercial cements in accordance with ISO 5833 standards [42]. Five cylindrical specimens for commercial and composite cements (12 mm and 6 mm in diameter) were prepared and tested using a mechanical testing machine (Syntech 10/D, MTS Corporation, Eden Prairie, MN, USA) at a crosshead speed of 20 mm/min. The average and standard deviation were calculated. The copper release from composite cements is a key factor in conferring antimicrobial and angiogenetic activities. Then, a leaching test was performed to assess the material’s ability to release Cu and to verify any difference in term of kinetics and the amount of copper between cements with different viscosities. Composite specimens were dipped in 60 mL of SBF maintained at 37 °C for 28 days. At fixed time frames (3 h, and 1, 3, 7, 14, and 28 days), an aliquot of the solution was chosen, diluted 1:10, and analysed with inductively coupled plasma mass spectrometry (ICP-MS, iCAP™ Q ICP-MS, Waltham, MA, USA), using bi-distilled water and SBF as the blank. The solution was not replaced by fresh SBF and the obtained results were calculated considering the decreasing volume of the solution and the performed dilution, thus obtaining a cumulative trend. The test was performed in triplicate.

### 2.3. Antibacterial Activity

#### 2.3.1. Bacteria Strain and Growth Conditions

The bacteria strain used for experiments was collected from a clinical isolate and tested for its multi-drug resistance (MDR) in the Clinical Microbiology Unit at the Novara Maggiore Hospital (Novara, Italy). The clinical isolate was obtained after patient’s informed consent in full accordance with the Declaration of Helsinki. Detailed information about the strain is reported in the Supplementary material. A single colony of an MDR *Staphylococcus epidermidis* (*S. epidermidis*) from an overnight culture on selective Trypticase Soy Agar plate (TSB, Sigma, Milan, Italy) was resuspended in 9 mL of Luria Bertani (LB) broth (Sigma, Milan, Italy) and incubated at 37 °C for 18 h. After incubation, a new fresh LB tube diluted 1:10 was prepared. The new tube was incubated at 37 °C for 3 h to determine the logarithmic growth phase. Finally, a fresh broth culture was prepared prior to each experiment by diluting bacteria in LB broth until the optical density (o.d.) was 0.001 at 600 nm, corresponding to a final concentration of 1 × 10^5^ cells/mL.

#### 2.3.2. *S. epidermidis* Biofilm Viability Evaluation

The antibacterial properties were evaluated using medium viscosity cements (CEMEX RX^®^-Cu) as the test composite, whereas cement containing undoped glass particles (CEMEX RX^®^-SBA3) was used as the control. Specimens were sterilized by means of temperature (3 h, 100 °C) prior to completing biological assays. Then, sterile specimens were placed in a 24 multiwell plate (Nunclon Delta Surface, Thermo Scientific, Waltham, MA, USA) and submerged with 1 mL of LB medium containing 1 × 10^5^ cells/mL, prepared as previously described. The plate was incubated for 90 min at 37 °C under agitation at 120 rpm to force biofilm cell adhesion onto the specimen surface (adhesion phase) [43,44,45,46]. Supernatants were then extracted to remove floating planktonic cells (separation phase) and specimens were gently washed 3 times with PBS to remove non-adherent cells [43,44,45,46]. Then, each specimen was rinsed with 1 mL of fresh LB medium and plate incubated for 1, 2, 3, 5, and 7 days at 37 °C for biofilm cultivation. At each time point, bacteria biofilm viability was evaluated by the colorimetric alamar blue assay (alamar Blue^®^, Thermo Fisher, Waltham, MA, USA) following the manufacturer’s instructions. Briefly, at each time-point, supernatants were removed from each well containing cells and replaced with alamar blue solution (10% *v*/*v* in fresh medium). Plates were incubated in the dark for 4 h and then 100 µL were removed, spotted into a new 96-well plate and fluorescence signals were evaluated with a spectrophotometer (Victor, Perkin Elmer, Waltham, MA, USA) at 590 nm. Experiments were performed in quadruplicate.

### 2.4. Statistical Analysis of Data

Data were analyzed using SPSS software (v20, IBM, New York, NY, USA) by means of one-way ANOVA followed by the Tukey test as a post-hoc analysis. Significance level was set at *p* < 0.05.

## 3. Results and Discussion

### 3.1. Bone Cement Physical Chemical Characterization

**Figure 1:**
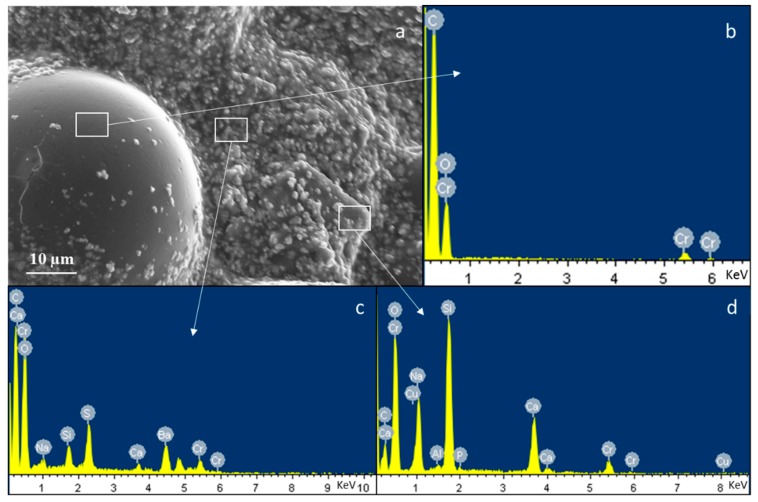
Field emission scanning electron microscopy (FESEM) and EDS analysis of high viscosity composite bone cement (CEMEX^®^ ISO-Cu); (**a**) morphology, (**b**) compositional analysis of PMMA sphere, (**c**) compositional analysis of BaSO_4_ and (**d**) compositional analysis of glass particles embedded in the polymeric matrix.

**Figure 2:**
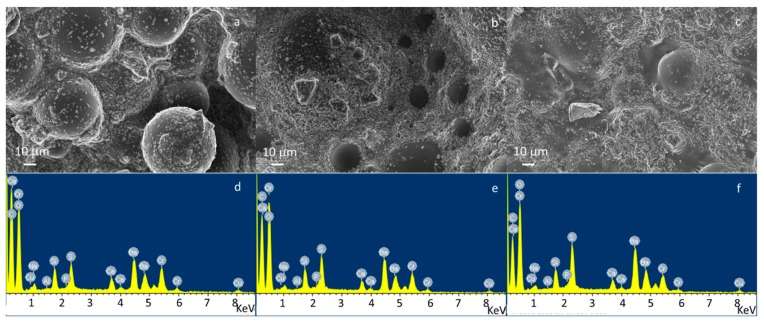
FESEM and energy dispersive X-ray spectroscopy (EDS) analysis of (**a**,**d**) CEMEX^®^ ISO-Cu, (**b**,**e**) CEMEX^®^ RX-Cu, and (**c**,**f**) CEMEX^®^ XL-Cu.

Morphological and compositional analyses of composite bone cements are reported in Figure 1 and Figure 2. The image and EDS in Figure 1 show the constituents of the composite sample at high viscosity as an example. Pre-polymerized PMMA sphere (Figure 1a,b), glass particles dispersed on a polymeric matrix (Figure 1a,d), and barium sulfate powders as the radio-opaque agent (Figure 1a,c). Figure 2 shows the micrographs and EDS analysis of a large area (about 1 mm^2^) of CEMEX^®^ ISO-Cu, CEMEX^®^ RX-Cu, and CEMEX^®^ XL-Cu. In Figure 2a–c, all samples show a PMMA sphere with variable dimensions of about 10–70 µm and BaSO_4_ powders that cover the surface of the cement and glass particles well dispersed in the polymeric matrix. EDS analysis of all composite cements confirmed the presence of glass exposed on the sample surface. The peaks of the elements characteristic of the glass (Na, Al, Si, P, Ca, and Cu) were clearly visible in all spectra, demonstrating that glass is well exposed on all cement surfaces. This is a fundamental aspect for imparting bioactive properties and allowing the release of copper ions and, as a consequence, inducing biological effects.

**Figure 3:**
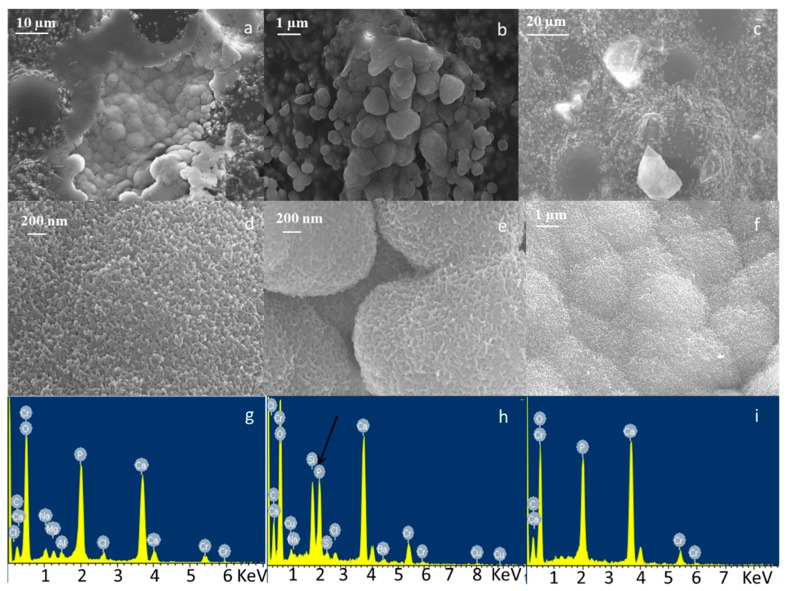
FESEM and EDS analysis of (**a**,**d**,**g**) CEMEX^®^ ISO-Cu, (**b**,**e**,**h**) CEMEX^®^ RX-Cu, and (**c**,**f**,**i**) CEMEX^®^ XL-Cu immersed in simulated body fluid (SBF) solution up to 7 days.

**Figure 4:**
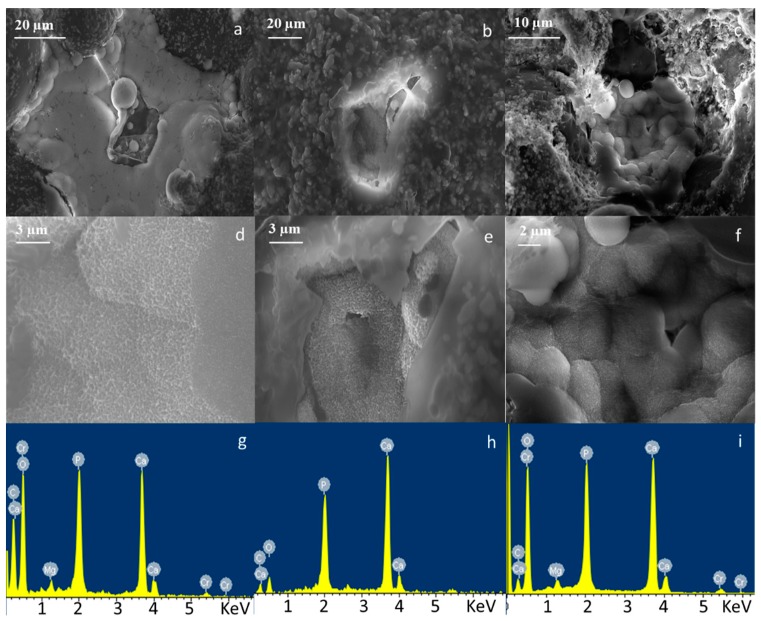
FESEM and EDS analysis of (**a**,**d**,**g**) CEMEX^®^ ISO-Cu, (**b**,**e**,**h**) CEMEX^®^ RX-Cu, and (**c**,**f**,**i**) CEMEX^®^ XL-Cu immersed in SBF solution up to 14 days.

**Figure 5:**
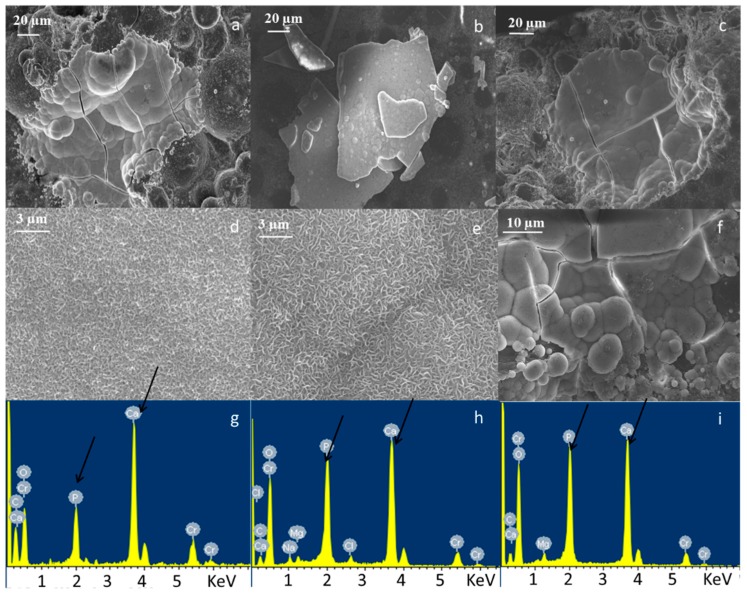
FESEM and EDS analysis of (**a**,**d**,**g**) CEMEX^®^ ISO-Cu, (**b**,**e**,**h**) CEMEX^®^ RX-Cu, and (**c**,**f**,**i**) CEMEX^®^ XL-Cu immersed in SBF solution up to 28 days.

Bioactivity testing was performed by immersing composite cements in SBF and evaluating the precipitation of HAp on their surface by means of FESEM-EDS analysis. Figure 3, Figure 4 and Figure 5 show the micrographs and corresponding EDS analysis of composite bone cements after 7, 14, and 28 days of SBF immersion, respectively. All composite cements evidenced the presence of agglomerates rich in Ca and P after seven days of immersion in SBF (Figure 3). The precipitates showed the typical globular morphology of in vitro-grown HAp (Figure 3d–f). At the beginning, they nucleated in the holes of the cement surface (Figure 3 and Figure 4) and then, after 14 days of SBF treatment, expanded across the whole surface (Figure 5).

**Figure 6:**
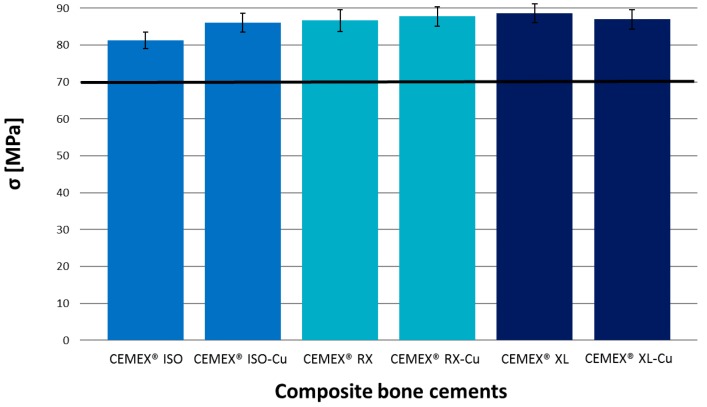
Compression strength evaluation of composite cements and commercial samples in accordance with ISO 5833-2002 standard. Bars represent means and standard deviations.

**Figure 7:**
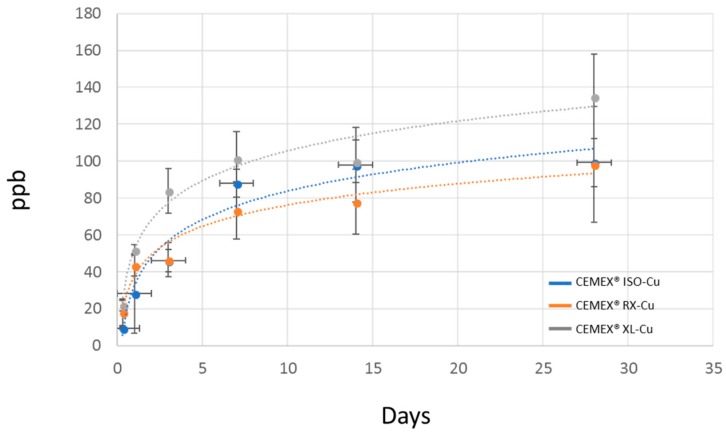
Copper release trend and amount for composite bone cements.

This result shows that SBA3-Cu glass powders were well dispersed and exposed on the composite cement surface, enabling the induction of nucleation and growth of HAp, which promoted the in vivo integration with the surrounding bone tissue. The pH values measured during the immersion in SBF remain in the range of physiological tolerability (7–7.8). After two days of SBF treatment, a moderate increase in pH (up to about 7.7) was observed, due to the release of alkaline cations from the surface of the glass into the solution during the first days [47]. Subsequently, pH values stabilized around 7.4. The presence and the release of copper did not appear to influence the bioactivity mechanism, as already observed by the authors in a previous work [22], which focused on the bioactivity evaluation of SBA3 doped with Cu in comparison to undoped glass. In this case, the viscosity of the cements also did not appear to influence the composite cement reactivity. The compression strength was evaluated according to ISO 5833-2002 [42], and the results are reported in Figure 6. All composite cements showed a compressive strength similar to the commercial control and higher than the limit value imposed by the ISO 5833 standard (70 MPa). The highest compressive strength (89 MPa) was reached by cements having very low viscosity, even though the values were not statistically different, as also observed by authors in other reports [39]. This preliminary mechanical characterization demonstrates that the addition of 10% inorganic phase did not modify the compression strength of cements, as already observed by authors for different composite cement compositions [37,38,39]. Thus, this provides an important starting point for future mechanical evaluation, such as bending and fatigue strength characterization. Leaching testing was performed to verify the composite cements’ ability to release copper ions and the possible influence of cement viscosity on the release kinetics. Figure 7 shows the trend in copper release. The release kinetics are comparable for all investigated composite cements. A considerable amount of copper ions was released in SBF solution during the first seven days of immersion. Subsequently, a lower copper release was observed up to 28 days without reaching a plateau. The obtained trend will allow the limiting of the incidence of infection both immediately after the surgical treatment and in the longer term. Considering the standard deviations, any significant difference in terms of copper amount was found among the CEMEX^®^ ISO-Cu, CEMEX^®^ RX-Cu, and CEMEX^®^ XL-Cu samples. The obtained results are encouraging since the maximum amount of released copper (about 130 ppb) was considerably lower than the exposure limit stated by the Environmental Protection Agency (EPA) for copper in drinking water (1.3 mg/L) [11]. Moreover, the obtained values were lower than the amount of Cu released from Cu-containing sol-gel bioactive glass, which did not show cytotoxic effect towards osteoblast-like cells (SaOS-2) [13].

### 3.2. Antibacterial Activity

Although questions remain about the involved mechanisms, copper clearly appears as an interesting candidate for the doping of surgically suitable materials. Accordingly, we decided to introduce copper-doped bioactive glass into commercial CEMEX^®^ bone cement to confer antibacterial properties. To test this hypothesis, an MDR *S. epidermidis* was selected as the test strain due to its high rate of orthopedic infections [43,48].

**Figure 8:**
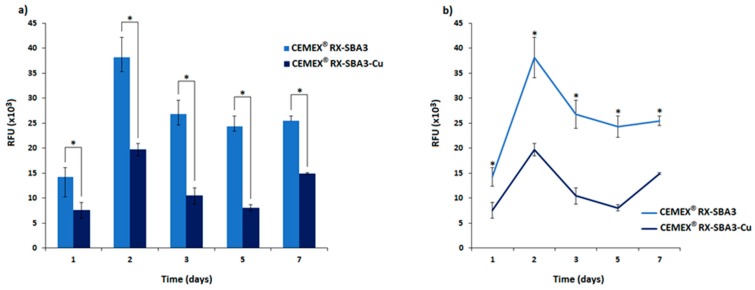
(**a**) CEMEX^®^ RX and CEMEX^®^ RX-Cu antibacterial activity towards *S. epidermidis* biofilm. The introduction of copper determined a statistically significant reduction in terms of bacteria viability that was observed at all the assayed time-points; *p* < 0.05, indicated by *. (**b**) Moreover, the copper activity resulted as stable in function of time; significant differences are denoted with *.

Results of the antibacterial activity of CEMEX^®^ RX-SBA3 and CEMEX^®^ RX-Cu specimens are reported in Figure 8. Viability is expressed in relative fluorescence units (RFU) according to alamar blue reading. In general, the introduction of copper conferred a marked antibacterial activity. By comparing the viability of the biofilm seeded onto the untreated CEMEX^®^ RX-SBA3 control and doped CEMEX^®^ RX-Cu, significant differences were observed at all the tested cements at one, two, three, five, and seven days (Figure 8a, *p* < 0.05, indicated by the *). Copper activity was observed during a relatively long time period of seven days (Figure 8b, significant differences marked by the *) where the viability of bacteria resulted in a range between 51.45 (±6.19) and 58.51% (±1.26) when normalized towards controls, considered as 100%. Detailed values are listed in Table 2. These data align with the findings related to copper release evaluation (Figure 7), where the CEMEX^®^ RX-Cu specimens showed a continuous and homogeneous release during the tested time-points.

The antimicrobial effect of copper-containing biomaterials was previously reported [49,50,51]. However, the investigated materials and the techniques used to introduce copper significantly differed from the material examined in this paper. The explanation for the copper efficacy is related to the evidence that copper is a toxic but essential trace element [3]. Notably, enzymes such as lysyl oxidase, tyrosinase, dopamine β-hydroxylase, cytochrome c oxidase, and super-oxide dismutase require copper as an electron donor/acceptor by alternating between the redox states Cu(I) and Cu(II) [52]. However, depending on the type of coordination of the copper to the protein, reactive hydroxyl radicals can be generated in a Fenton-type reaction. These extremely reactive hydroxyl radicals can participate in a number of reactions detrimental to cellular molecules, such as the oxidation of proteins and lipids [53].

Despite the strong antibacterial activity demonstrated by copper, some bacteria have evolved mechanisms to protect themselves from it. For example, Rensing et al. [54] showed that *E. coli* was able to convert the ATPase-dependent CopA transporter to pump excess Cu(I) from the cytoplasm to the periplasm.

Accordingly, the antibacterial results obtained here can be considered as a promising starting point, to insert copper into glass and develop composite cements; however, the antimicrobial effect can be improved by tailoring the parameters of the ion-exchange process and the amount of inorganic phase in the PMMA matrix. Even if copper doping resulted in approximately 50% bacteria inhibition, the remaining 50% could proliferate and propagate the infection despite the release of Cu ions. Nevertheless, the in vivo combination of a bioactive surface and the release of an antimicrobial agent that is able to limit the bacteria proliferation can direct the “race for the surface” toward the host cell adhesion and therefore promote the material integration. For the discussed reasons, more studies related to the amount of copper release and the use of different strains are required to achieve results that can be translated to a possible application.

## 4. Conclusions

PMMA-based composite bone cements were developed by incorporating Cu-doped bioactive glass particles into commercial formulations with three different viscosities: high, low, and very low. A multifunctional material was created by adding a single inorganic phase.

All composite cements showed homogenous glass distribution in the polymeric matrix and glass exposure on cement surface. The glass exposure conferred bioactive properties to composite samples. After seven days of SBF immersion the presence of agglomerates rich in Ca and P, the typical globular morphology of in vitro grown HAp was observed on samples surface. All composite cements were released copper ions with kinetics potentially useful to fight infection development. Moreover, the preliminary antibacterial test on CEMEX^®^RX-Cu demonstrated the ability of the released copper to reduce the viability of the *S. epidermidis* biofilm. Finally, the introduction of glass powders did not alter the compression strength of the cement. The viscosity of the used commercial formulation did not influence the glass dispersion in the PMMA matrix; therefore, no significant difference was observed in terms of bioactivity, and leaching and mechanical properties among CEMEX^®^ ISO-Cu, CEMEX^®^ RX-Cu, and CEMEX^®^ XL-Cu samples.

The obtained data represent a promising starting point for the realization of innovative, multifunctional, and antibiotic-free PMMA-based bone cement that is both osteoinductive and antibacterial. Future investigations will be performed to estimate the cytocompatibility of the composites and to examine their antimicrobial properties.

## Figures and Tables

**Table 1:** Test materials and related compositions.

Sample Name	Solid Polymer (wt %)	BaSO_4_ (wt %)	SBA3 0.05 M (wt %)	Solid Polymer/MMA	Viscosity
CEMEX^®^ ISO	84.30	13	0	3:1	High
CEMEX^®^ ISO-Cu	74.30	13	10	3:1	High
CEMEX^®^ RX	88.27	9	0	3:1	Low
CEMEX^®^ RX-Cu	78.27	9	10	3:1	Low
CEMEX^®^ XL	85	12	0	3:1	Very low
CEMEX^®^ XL-Cu	75	12	10	3:1	Very low

**Table 2:** Bacteria cultivated onto copper-doped specimens (CEMEX^®^ RX-SBA3-Cu) % of viability in comparison with untreated control (CEMEX^®^ RX-SBA3). Data are expressed as means ± standard deviation.

Sample	Day 1	Day 2	Day 3	Day 5	Day 7
CEMEX^®^ RX-SBA3	*(cnt = 100%)*				
CEMEX^®^ RX-SBA3-Cu	52.95 (±21.27)	51.45 (±6.19)	38.96 (±15.59)	33.09 (±7.4)	58.51 (±1.26)

## Supplementary Materials

The following are available online at http://www.mdpi.com/1996-1944/11/6/961/s1, Table S1: *S. epidermidis* broth dilution susceptibility test results. Susceptibility pattern was expressed as follow: S: Susceptible, I: Intermediate, R: Resistant. MIC represents minimal inhibitory concentration.
