# Supplementary material for: Copper-Doped Bioactive Glass as Filler for PMMA-Based Bone Cements: Morphological, Mechanical, Reactivity, and Preliminary Antibacterial Characterization
Source: Materials (Basel). 2018 Jun 6;11(6):961. doi: 10.3390/ma11060961 (PMC6025430; doi:10.3390/ma11060961)
Supplement: Supplementary file 1 [file materials-11-00961-s001.pdf]

Supplementary Material

Copper-doped bioactive glass as filler for PMMA-based bone cements: morphological, mechanical, reactivity and preliminary antibacterial characterization

S1. S. epidermidis clinical isolate strain details

Bacteria strain used for experiments was collected from clinical isolate and tested for its multi-drugs resistance (MDR) by the Clinical Microbiology Unit at the Novara Maggiore Hospital. Clinical isolate was collected from blood. Strain drug resistance has been assayed by the Clinical Microbiology Unit using the broth dilution susceptibility test. Results are summarized in Table 1.

Table 1. *S. epidermidis* broth dilution susceptibility test results. Susceptibility pattern was expressed as follow: S: Susceptible, I: Intermediate, R: Resistant. MIC represents minimal inhibitory concentration.

**Antimicrobial**	**Susceptibility**	**MIC (μg/ml)**
Ampicillin	R	8
Cefazolin	R	≤8
Chloramphenicol	S	≤8
Ciprofloxacin	R	>2
Clindamycin	R	>2
Gentamicin	R	>8
Levofloxacin	S	≤2
Ofloxacin	I	4
Oxacillin	R	>2
Tetracycline	S	≤4
Vancomycin	S	≤2
